# Flow Cytometric Analysis of Leukocyte Populations in the Lung Tissue of Dromedary Camels

**DOI:** 10.3390/vetsci9060287

**Published:** 2022-06-10

**Authors:** Jamal Hussen, Turke Shawaf, Naser Abdallah Al Humam, Sameer M. Alhojaily, Mohammed Ali Al-Sukruwah, Faisal Almathen, Francesco Grandoni

**Affiliations:** 1 Department of Microbiology, College of Veterinary Medicine, King Faisal University, Al-Ahsa 31982, Saudi Arabia; nalhumam@kfu.edu.sa (N.A.A.H.); 219028688@student.kfu.edu.sa (M.A.A.-S.); 2 Department of Clinical Sciences, College of Veterinary Medicine, King Faisal University, Al-Ahsa 31982, Saudi Arabia; tshawaf@kfu.edu.sa; 3 Department of Biomedical Sciences, College of Veterinary Medicine, King Faisal University, Al-Ahsa 31982, Saudi Arabia; salhojaily@kfu.edu.sa; 4 Agricultural and Veterinary Training and Research Station, King Faisal University, Al-Ahsa 31982, Saudi Arabia; 5 Department of Public Health, College of Veterinary Medicine, King Faisal University, Al-Ahsa 31982, Saudi Arabia; falmathen@kfu.edu.sa; 6 The Camel Research Center, King Faisal University, Al-Ahsa 31982, Saudi Arabia; 7 Research Centre for Animal Production and Aquaculture, Consiglio per la Ricerca in Agricoltura e l’analisi dell’economia Agraria (CREA), Via Salaria, 31-Monterotondo, 00016 Rome, Italy; francesco.grandoni@crea.gov.it

**Keywords:** immune cells, lung, camel, flow cytometry, phagocytosis, mucosal immunology

## Abstract

Respiratory tract infections are among the most common infections in dromedary camels, with a high impact on animal health, production, and welfare. Tissue-specific distribution of immune cells is one of the important factors that influence the nature and outcome of the immune response to pathogens. Several protocols have recently been described for the flow cytometric analysis of immune cells in the lung tissue of several species. However, no such protocol currently exists for dromedary camels. The aim of the present study was, therefore, to establish a flow cytometric protocol for the identification of immune cell populations in the camel lung tissue and the evaluation of some of their phenotypic and functional properties. Combined staining of camel lung leukocytes with monoclonal antibodies to the pan-leukocyte marker CD45 and the myeloid cell marker CD172a allowed the identification of myeloid cells (CD45^+^CD172a^+^) and lymphoid cells (CD45^+^CD172a^−^) in the lung of healthy camels. The cell adhesion molecules CD11a and CD18 were found in a higher abundance on myeloid cells compared to lymphoid cells. Based on their differential expression of the LPS receptor CD14, macrophages (CD172a^+^CD14^high^ cells) were identified as the most abundant immune cell population in the camel lung tissue. In contrast to their dominance in camel peripheral blood, granulocytes (CD172a^+^CD14^low^) presented only a minor population in the lung tissue. The higher frequency of γδ T cells in the lung tissue than in peripheral blood suggests a role for these cells in the pulmonary immune system. Flow cytometric analysis of bacterial phagocytosis and ROS production upon bacterial stimulation revealed high antimicrobial activity of camel lung phagocytes, which was comparable with the antimicrobial activity of blood granulocytes. Comparative analysis of immune cell distribution between the cranial and caudal lobes of the camel lung revealed a higher frequency of granulocytes and a lower frequency of macrophages in the cranial compared to the caudal lung lobe. In addition, the higher frequency of cells expressing the M2 macrophage marker CD163 in the caudal lung tissue, with a slightly higher fraction of MHCII-positive cells (M1 phenotype) in the cranial lung tissue, may suggest the distribution of different macrophage subtypes in the different lobes of the camel lung. Such differences between lung lobes could influence the effectiveness of the immune response to infection or vaccination with respiratory pathogens. Collectively, the present study identified some similarities and differences between camels and other farm animals regarding the distribution of the main immune cell populations in their lungs. Further studies are required for comprehensive immunophenotyping of the cellular pulmonary immune system in camels.

## 1. Introduction

The respiratory tract is a major gate for pathogens that can result in life-threatening diseases. Therefore, various defense strategies have evolved in the respiratory tract to control pathogen replication and regulate inflammatory responses [1]. Effective pulmonary immune responses to infection or injury require an intact and highly regulated immune system, while a dysregulated immune system is frequently associated with chronic respiratory disease [1,2]. The presence of distinct cell populations and their specific phenotype is a key measure of successful immune responses in the lung [3]. Flow cytometry provides an important methodology that allows detailed identification of the composition, phenotype, and function of cells of the immune response [4,5]. It has proven effective in characterizing cells in several bodily fluids and organ systems [6,7,8,9,10].

The cellular immune system of the lung includes cells of the mononuclear phagocytes system, polymorphonuclear granulocytes, and lymphocytes [1,2]. Macrophages are the predominant immune cell type of the respiratory tract, with key roles during infection and inflammation. They contribute to the lung defense system through their innate sensing capacity, antimicrobial activity (phagocytosis and killing of pathogens), scavenging foreign material and cellular debris, antigen-presentation to T cells and activation of the adaptive immune response, clearing apoptotic and necrotic cells and resolution of inflammation, and production of several immune mediators, including cytokines and chemokines [6,11,12]. In addition, phenotypic and functional plasticity is one of the remarkable characteristics of macrophages. In vitro differentiation of monocyte-derived macrophages may result in the polarization toward an inflammatory M1 (IFN-γ/classically activated) or anti-inflammatory M2 (IL-4/alternatively activated) phenotype with different roles in infection immunity [13]. The in vivo relevance of this classification is, however, still controversial due to the complexity of the polarizing signals in the respective disease situation and tissue microenvironment [14,15].

Neutrophils are one of the most abundant leukocyte populations in blood, with a primary role in pathogen sensing, killing, and removal [3]. In response to acute stimulation with pathogen-associated molecular patterns, danger-associated molecular patterns, or inflammatory cytokines and chemokines, neutrophils are the first immune cell attracted to the lung [8], followed by monocytes and lymphocytes, which predominate the chronic phase of inflammation [9,10].

Marker surface antigens expressed by myeloid immune cells in the lung include CD172a, CD14, CD163, and major histocompatibility complex (MHC) class II molecules. The signal regulatory protein α (SIRP α) is an inhibitory protein that mediates a so-called ‘do not eat me’ signal, suppressing the phagocytosis activity of alveolar macrophages through interaction with surfactant proteins [16], an effect that can be overcome by SIRPα downregulation upon LPS/TLR4 interaction in macrophages [17]. CD14 is a well-known receptor for the cell-wall component of Gram-negative bacteria and lipopolysaccharide (LPS), and it is mainly expressed on macrophages and monocytes [18]. CD163, which is exclusively expressed by macrophages and monocytes, is the receptor for the binding and uptake of hemoglobin–haptoglobin complexes [19]. MHC II molecules are antigen receptors involved in the presentation of exogenous peptide antigens to the T cell receptor and the subsequent activation of antigen-specific T helper cells [20,21].

Several protocols have recently been described for flow cytometric analysis of immune cells in human [2], rhesus [22], murine [23], bovine [24,25], porcine [26], equine [27], and canine [28] lung tissues. However, no such protocol currently exists for the dromedary camel. The aim of the present study was, therefore, to establish a flow cytometric protocol for the identification of immune cell populations in the camel lung tissue and the evaluation of some of their phenotypic and functional properties. The availability of such a protocol would lead to an improved understanding of immune mechanisms in the respiratory tract and other mucosal systems.

## 2. Materials and Methods

### 2.1. Ethical Approval

The Ethics Committee at King Faisal University, Saudi Arabia, approved all the experimental procedures used in this study (Permission number KFU-REC/2019-10-01).

### 2.2. Materials

The RPMI medium and the Fluoresceinisothiocyanate were acquired from Sigma-Aldrich (FITC, Sigma-Aldrich, St. Louis, MO, USA). The vacutainer tubes containing EDTA were acquired from Becton Dickinson (BD Heidelberg, Germany. The antibodies used for cell labeling are described in Table 1. The anti-CD14 antibody and the goat anti-mouse secondary antibodies were acquired from Thermofisher Scientific (Thermofisher Scientific, Hennigsdorf, Germany. The anti-MHCII and anti-CD163 antibodies were acquired from Kingfisher Biotech (Kingfisher Biotech, Saint Paul, Minnesota, USA). The antibodies to CD172a and CD11a were purchased from the monoclonal antibody center of the Washington State University (WSU, Pullman, Washington, USA). The anti-CD4 and anti-WC1 antibodies were acquired from XCeltis (XCeltis GmbH, Mannheim, Germany). Heat-killed staphylococcus aureus was purchased from Calbiochem (Pansorbin, Calbiochem, Merck, Nottingham, UK). Dihydrorhodamine-123 was acquired from Mobitec (Mobitec, Goettingen, Germany). The Accurie C6 flow cytometer was acquired from BD Biosciences (BD Biosciences, San Jose, CA, USA).

### 2.3. Animals and Clinical Examination

The present study was conducted in vitro using blood and lung tissue samples collected from five clinically healthy dromedary camels. The animals were non-pregnant and non-lactating female dromedary camels from the Majaheem breed aged between 9 and 12 years. The camels were selected from animals brought to the Al-Omran slaughterhouse in the Al-Ahsa region in Saudi Arabia. The identification of healthy animals was based on clinical respiratory scoring, with no abnormal respiratory signs such as cough, nasal discharge, dyspnea, or lung sounds. The animals were also free from other clinical infections including mastitis, metritis, or joint disorders. In addition, the camels were tested negative for the zoonotic virus Middle East respiratory syndrome coronavirus (MERS-CoV; using the BIONOTE ^®^ Rapid MERS-CoV Ag Test Kit (BioNote Inc, Hwaseong, Gyeonggi, Republic of Korea) using nasal swabs [29]. Lung tissue samples were obtained from the cranial and caudal lobes of each slaughtered animal. Immediately post-mortem, the lungs of each camel were inspected for abnormal lesions characteristic of respiratory infections before further processing. Next, approximately 2 cm^3^ of tissue was dissected from the cranial and caudal lobes of the right lung and placed in a sterile tube containing RPMI medium.

### 2.4. Blood Sampling and Cell Separation

Blood samples were collected from all animals by puncture of the vena jugularis externa using vacutainer EDTA tubes. Leukocytes were separated after removal of red blood cells (RBCs) by repeated cycles of hypotonic lysis [21]. After dilution with PBS (1:9) in 15 mL falcon tubes, blood samples were centrifuged at 4 °C and 1000× *g* for 25 min without break. After plasma removal, the red blood cells were lysed using 20 s incubation in 5 mL distilled water followed by the addition of 5 mL 2 × PBS and a 10 min centrifugation step at 500× *g* and 4 °C. After re-suspending the cells, the RBC lysis was repeated until the collection of a clear white pellet. Subsequently, 10 mL PBS were added to the cells followed by two washes (250× *g* and 100× *g* for 10 min each). Finally, the cells were adjusted to 5 × 10^6^ cells/mL in RPMI medium. For lung cell separation, collected tissues were minced and incubated in RPMI medium supplemented with 100 IU/mL penicillin-streptomycin (all from Invitrogen, Manchester, UK), and containing 2 mg/mL collagenase D and 0.05 mg/mL DNase I (both from Roche). The suspension was passed through a 40 μm filter, and RBCs were lysed by hypotonic lysis. After a final wash with PBS, the cell count was estimated using Neubauer’s counting hemocytometer and light microscopy. The cell suspension was adjusted to 5 × 10^6^ cell/µL RPMI cell culture medium.

### 2.5. Monoclonal Antibodies

The antibodies used for labeling the cells are showed in Table 1. All antibodies have been tested for reactivity against camel leukocytes in previous studies [30,31,32,33,34].

### 2.6. Cell Labeling and Flow Cytometry

Cell labeling of lung and blood leukocytes with monoclonal antibodies was performed as previously described [10,35]. Separated lung cells or blood leukocytes (1 × 10^6^/well in 100 µL staining buffer) were incubated for 20 min at 4 °C with the following combinations of unlabeled primary monoclonal antibodies to cell surface antigens of camel leukocytes (mAbs; Table 1): CD45/CD172a, CD163/MHC-class II, or CD4/WC1 or with a combination of anti-CD11a-PE and anti-CD18-FITC mAbs. After two washing steps (by adding 150 µL washing buffer followed by centrifugation at 300× *g* for 3 min), the cells were stained with fluorochrome-labeled antibodies to mouse IgG1, IgG2a, and IgM isotypes for 20 min at 4 °C. In a third staining step, PerCP-labeled monoclonal antibodies to CD14 were added to the cells stained with CD45 and CD172a. Staining with mouse isotype control antibodies was also performed. After the final cell wash, the Acuri C6 flow cytometer (Accuri C6, Becton Dickinson Biosciences) was used for the acquisition of at least 100,000 total cells. CFlow Software, Version 1.0.264.21 (BD Biosciences) was used for data analysis.

### 2.7. Analysis of Phagocytosis Activity

Bacterial phagocytosis by lung or blood phagocytes was analyzed by flow cytometry, as previously described [36]. Labeling of heat-killed *staphylococcus aureus* with fluoresceinisothiocyanate (FITC) was performed according to manufacturer instructions. Lung or blood leukocytes (1 × 10^5^/well in RPMI medium) were incubated with FITC-labeled *S. aureus* (40 bacteria/cell) in 96-well plates for 45 min at 37 °C. The percentage of phagocytic cells (cells with elevated FITC signal) was calculated after cell acquisition by the Acuri C6 flow cytometer.

### 2.8. Measurement of Reactive Oxygen Species Production

Reactive oxygen species (ROS) generation by lung or blood phagocytes was analyzed by flow cytometry as previously described [36]. Lung or blood leukocytes (1 × 10^5^/well in RPMI medium) were stimulated with killed *S. aureus* bacteria or left without stimulation in 96-well plates (20 min; 37 °C). ROS production was detected using the addition of 0.5 µg/mL dihydrorhodamine-123 (DHR-123) to the cells. Finally, cells were washed with PBS (300× *g* for 3 min) and the ROS production was evaluated by estimating the green fluorescence intensity of cells by flow cytometry.

### 2.9. Statistical Analyses

Statistical analysis was performed using the Microsoft Excel program (Microsoft Office 2016) and the statistical software program Prism (GraphPad software version 5, GraphPad Software, San Diego, CA, USA). The results were presented as scattered dot plots with means ± standard error of the mean (SEM). Data normal distribution was evaluated using the Kolmogorov–Smirnov test (with the Dallal–Wilkinson–Lilliefor *p*-value). The one-way analysis of variance (ANOVA) or Friedman test was used to compare the means for normally distributed data or for data that failed to pass the normality test, respectively. Differences between the mean were considered significant if the *p*-value was less than 0.05.

## 3. Results

### 3.1. The Identification of Main Leukocyte Populations in the Lung Tissue of Camels

**Figure 1:**
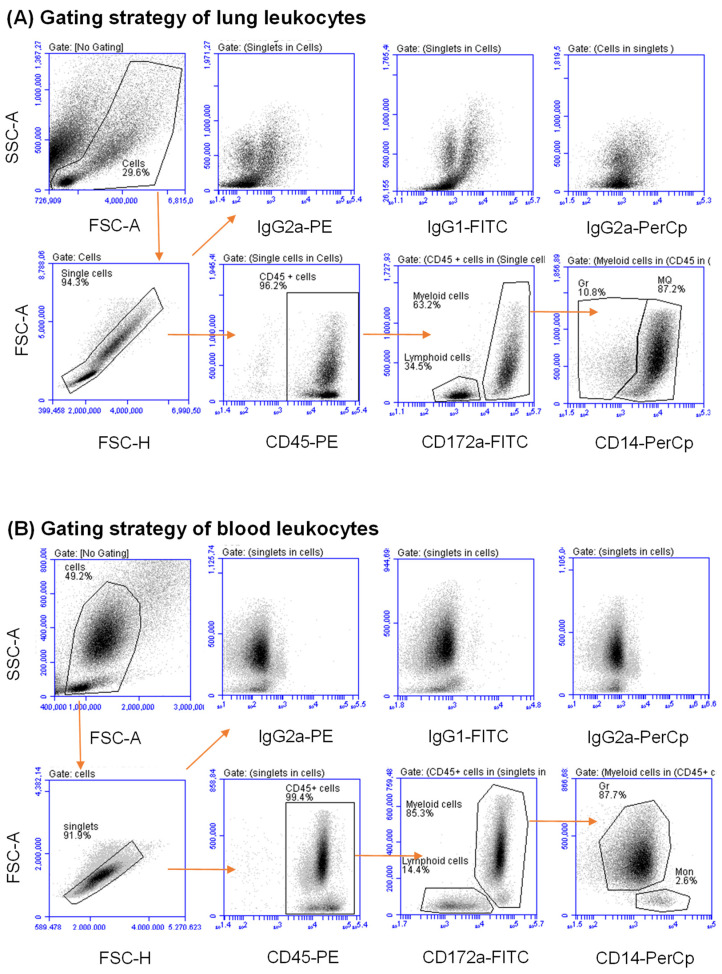
Flow cytometric analysis of leukocyte populations in the lung tissue and peripheral blood of dromedary camels. Lung cells (**A**) and blood leukocytes (**B**) were labeled with monoclonal antibodies to CD45, CD172a, and CD14 or with isotype control antibodies, and labeled cells were analyzed by flow cytometry. After the exclusion of cell debris in a SSC/FSC dot plot, a gate was set on single cells in a FSC-H against FSC-A dot plot. Total lung and total blood leukocytes were identified as CD45-positive cells. Lung and blood myeloid cells were differentiated from lymphoid cells based on their expression level of the myeloid marker CD172a. Lung macrophages and blood monocytes were identified based on their higher expression of the monocytic marker CD14 (CD172a^high^CD14^high^), while granulocytes were identified as CD172a^high^CD14^low^ cells.

Cell staining with mAb to CD172a enabled the identification of myeloid (CD45^+^CD172a^+^) and lymphoid (CD45^+^CD172a^−^) cells within CD45-positive lung and blood leukocytes (Figure 1A,B). The differential expression of the LPS receptor CD14 identified lung macrophages and blood monocytes as CD172a^+^CD14^high^ cells, while granulocytes were CD172a^+^CD14^low^ cells (Figure 1A,B).

**Figure 2:**
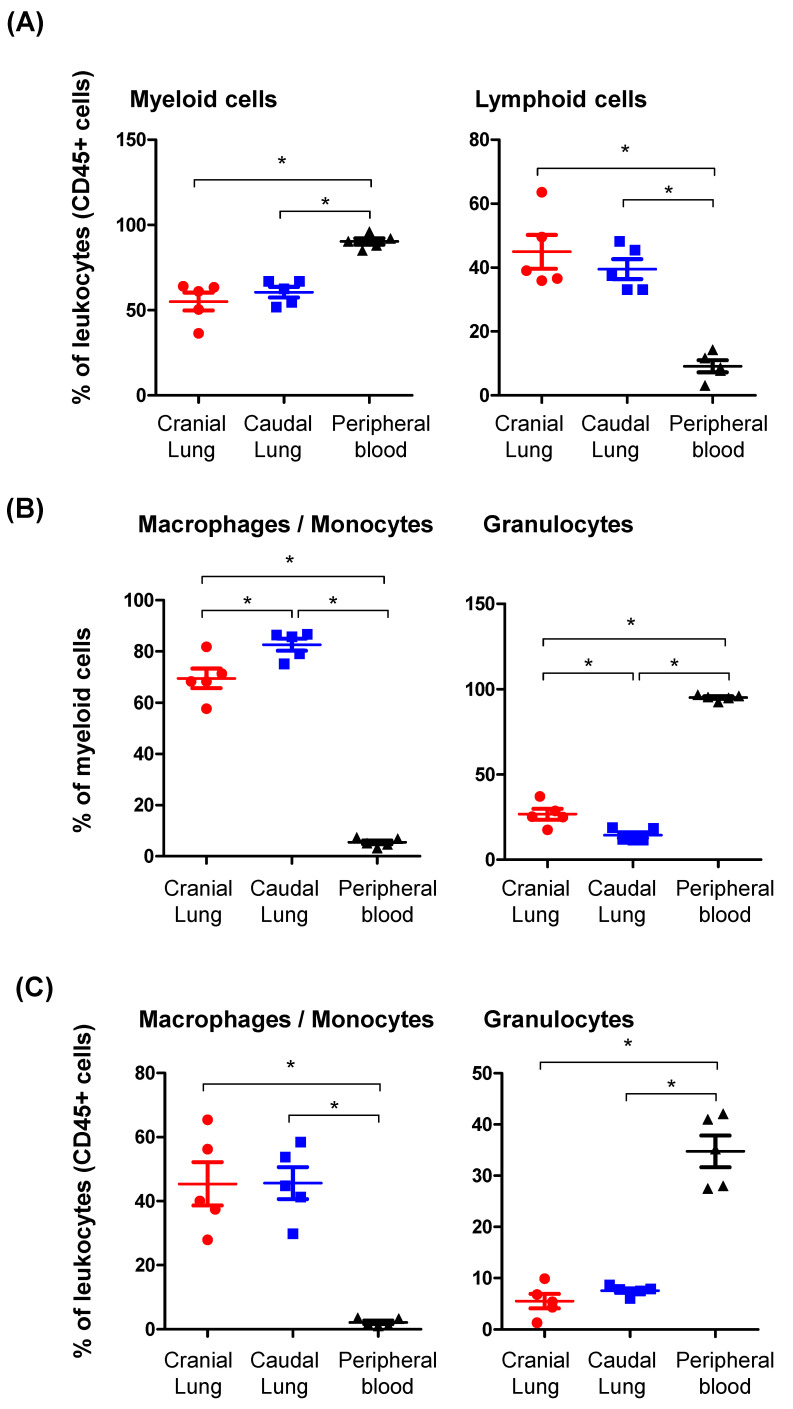
Leukocyte populations in cranial and caudal lung tissue and the peripheral blood of dromedary camels. Camel lung and blood leukocytes were labeled with antibodies to CD45, CD172a, and CD14, and labeled cells were analyzed by flow cytometry. (**A**) The percentage of myeloid (CD172a-positive cells) and lymphoid (CD172a-negative cells) cells within total leukocytes (CD45-positive cells) were calculated and presented graphically for cranial and caudal lung tissues and blood leukocytes. (**B**) Lung macrophages and blood monocytes were differentiated from granulocytes based on the differential expression of CD14. The percentages of macrophages/monocytes and granulocytes within lung and blood myeloid cells were calculated and presented graphically for cranial and caudal lung tissue and blood leukocytes. (**C**) The percentages of macrophages/monocytes and granulocytes within total lung and blood leukocytes were calculated and presented graphically for cranial and caudal lung tissue and peripheral blood. * indicates a significant difference between the groups (*p* < 0.05).

In comparison to their composition in peripheral blood, the leukocyte population in the lung tissue contained a lower percentage of myeloid cells (57.7 ± 3.1% versus 90.3 ± 1.9 of total CD45^+^ cells in the blood) and a higher percentage of lymphoid cells (42.2 ± 3.5% versus 9.1 ± 1.8% of total CD45^+^ cells in the blood) (Figure 2A). There were no significant differences between the cranial and caudal regions of the lung regarding the distribution of myeloid and lymphoid cells (*p* > 0.05). Within the myeloid cell population, the percentage of macrophages/monocytes was significantly higher in lung tissue (76.1 ± 3.8% of CD172a^+^ cells) than in peripheral blood (5.4 ± 0.8% of CD172a^+^ cells). In contrast, lung myeloid cells contained only a lower fraction of granulocytes (20.4 ± 0.8% of CD172a^+^ cells), when compared to peripheral blood (95.2 ± 0.7% of CD172a^+^ cells) (Figure 2B,C). The comparison between the cranial and caudal regions of the lung tissue revealed a lower frequency of macrophages (69.5 ± 3.9% versus 82.6 ± 2.3% of myeloid cells in the cranial lung tissue) but a higher frequency of granulocytes (26.4 ± 3.5% versus 15.0 ± 2.1% of myeloid cells, when compared to the caudal lung tissue (*p* < 0.05) (Figure 2B). However, the differences were not significant when comparison was made between the percentages of macrophages or granulocytes within the whole leukocyte population (*p* > 0.05) (Figure 2C).

### 3.2. Expression Pattern of CD163 and MHCII on Camel Lung Leukocytes

**Figure 3:**
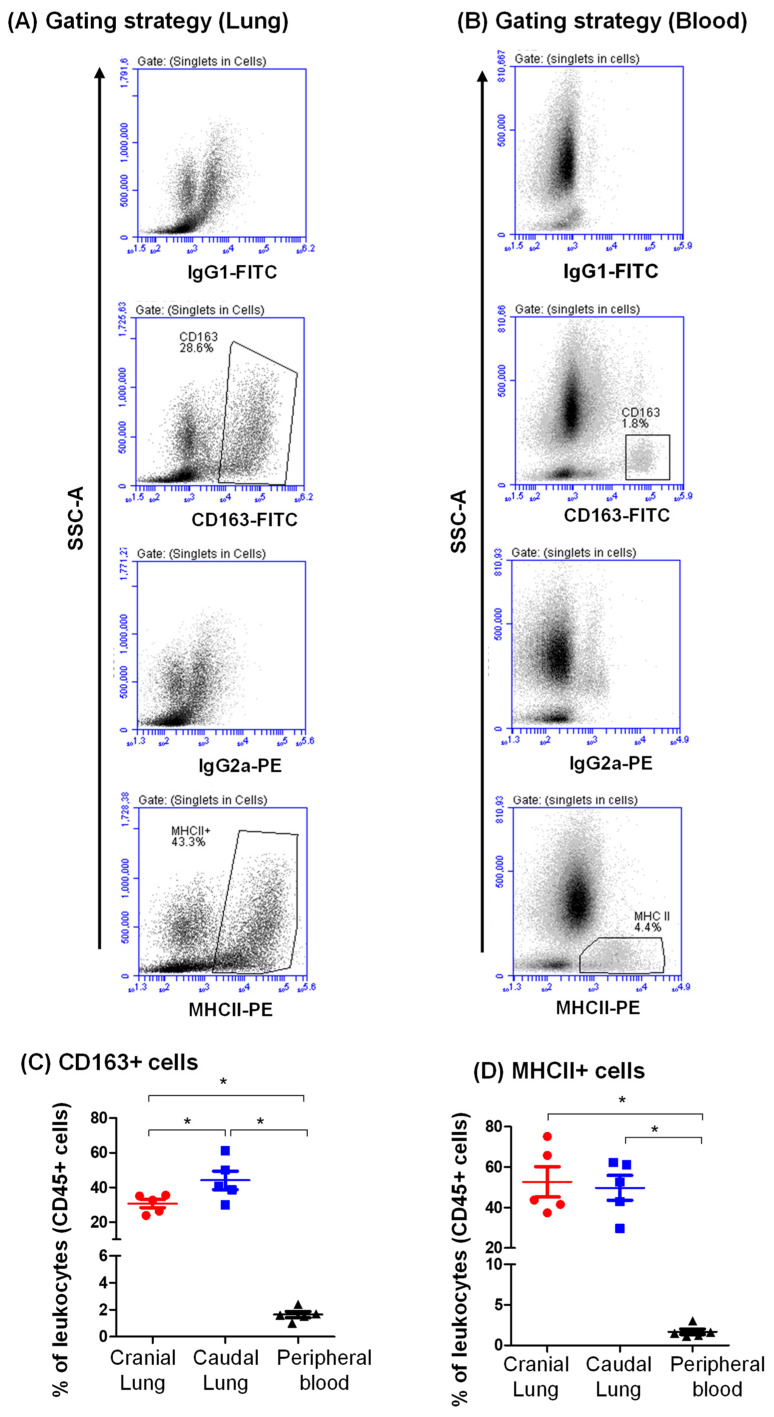
Flow cytometric analysis of the expression level of CD163 (**A**) and MHCII (**B**) on lung and blood leukocytes. (**A**) Lung cells and blood leukocytes were labeled with monoclonal antibodies to CD163 and MHCII molecules or with isotype control antibodies, and labeled cells were analyzed by flow cytometry. The percentage of CD163-positive cells within lung cells and blood leukocytes was identified in an SSC against CD163 dot plot. The percentage of MHCII-positive cells within lung cells and blood leukocytes was identified in an SSC against MHCII dot plot. (**C**) Data of CD163 (**C**) and MHCII (**D**) expression were presented graphically for cranial and caudal lung tissue and peripheral blood leukocytes. * indicates a significant difference between the two groups (*p* < 0.05).

Staining with antibodies to CD163 identified a large population of lung macrophages (37.4 ± 3.2% of leukocytes), which was higher in frequency (*p* < 0.05) than the fraction of CD163-positive monocytes in the blood (1.6 ± 0.2% of blood leukocytes) (Figure 3A–C). The percentage of CD163 cells was significantly higher in the caudal (42.9 ± 6.2% of total CD45^+^ cells) than in the cranial (31.9 ± 1.7% of total CD45^+^ cells) tissue (Figure 3A–C). Antibodies to MHCII molecules stained a major population of lung leukocytes (51.3 ± 6.5% of total CD45^+^ cells) with a slightly (*p* > 0.05) higher fraction of MHCII-positive cells in the cranial (52.7 ± 7.5% of total CD45^+^ cells) than in the caudal (49.8 ± 6.1% of total CD45^+^ cells) lung tissue. The percentage of MHCII-positive cells within blood leukocytes (1.7 ± 0.3% of blood leukocytes) was significantly lower than in the lung (Figure 3A–C).

### 3.3. Identification of CD4-Positive Helper T Cells and Gd T Cells in the Camel Lung

**Figure 4:**
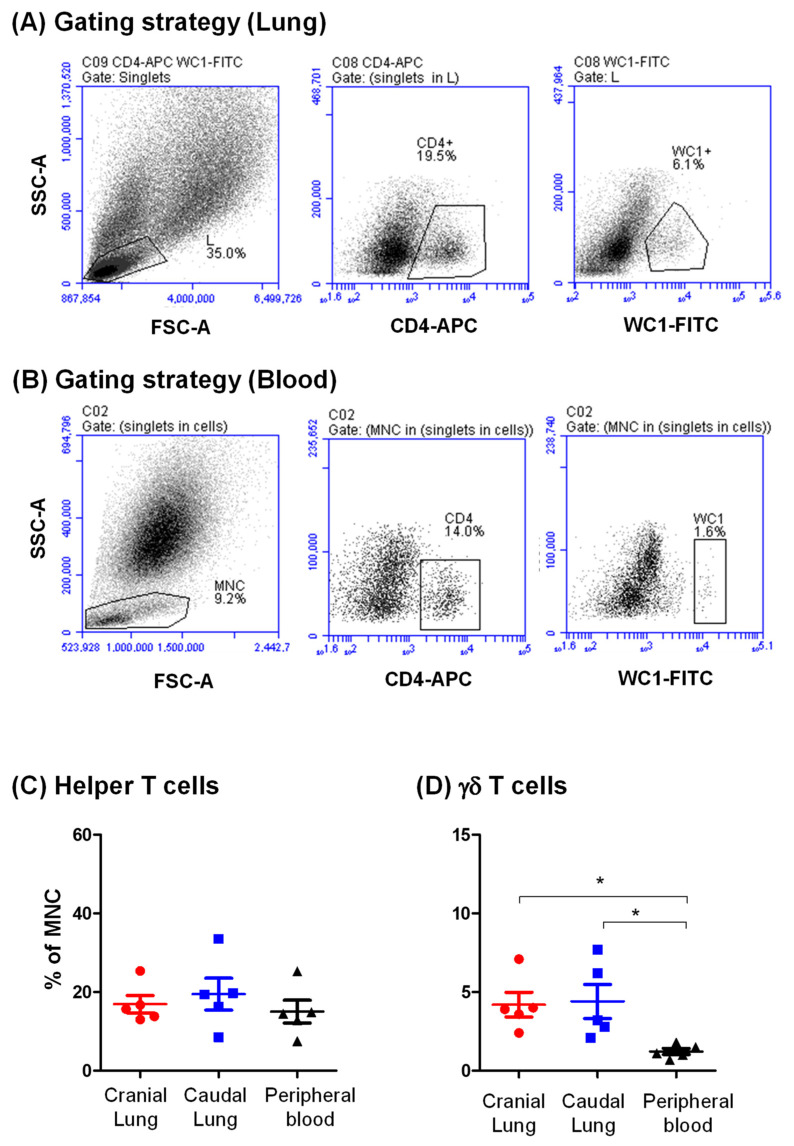
Flow cytometric analysis of selected lymphocyte subsets in the lung tissue and peripheral blood of dromedary camels. Lung cells or blood leukocytes were labeled with monoclonal antibodies to CD4 and WC1, and labeled cells were analyzed by flow cytometry. After setting a gate on lung lymphocytes (**A**) and blood mononuclear cells (**B**), the CD4-positive T helper cells and WC1-positive γδ T cells were identified based on their higher expression of the marker molecules. Data for cranial and caudal lung tissue and blood leukocytes were calculated and presented graphically for helper T cells (**C**) and γδ T cells (**D**). * indicates a significant difference between the two groups (*p* < 0.05).

The staining of lung cells with mAbs to CD4 molecules labeled 18.2 ± 3.1% of lung mononuclear cells showed comparable values for cranial and caudal lung tissues (*p* > 0.05). Similarly, the percentage of CD4-positive T cells within blood mononuclear cells did not differ significantly from that in the lung (15.0 ± 2.9% of blood mononuclear cells) (Figure 4A–C). The antibody to the bovine workshop cluster 1 (WC1) antigen identified a population of lung mononuclear cells (4.4 ± 1.0%), which was significantly higher (*p* < 0.05) than the WC1-positive population within blood mononuclear cells (1.2 ± 0.2%) (Figure 4B,D).

### 3.4. Cell Adhesion Molecules Expression on Lung Leukocytes

**Figure 5:**
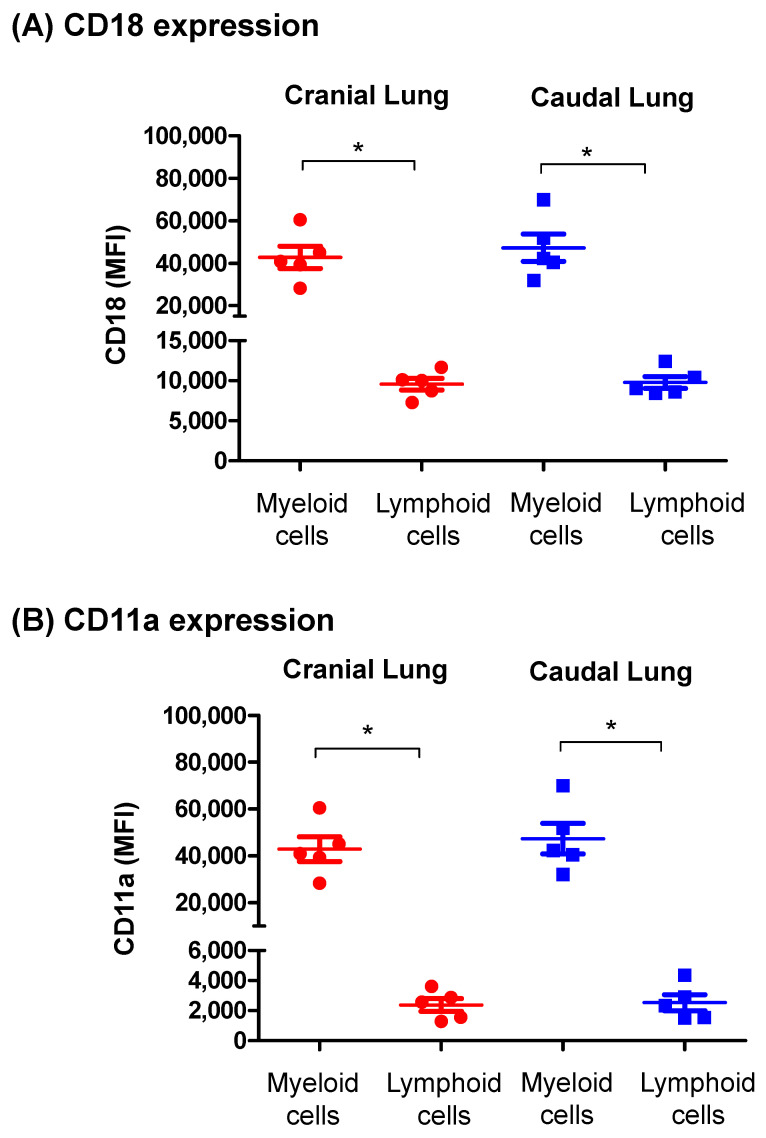
The expression level of the cell adhesion molecules CD18 (**A**) and CD11a (**B**) on camel lung leukocytes. Lung cells were labeled with anti-CD18-FITC and anti-CD11a-PE antibodies and analyzed by flow cytometry. CD18 and CD11a abundance was measured as the mean fluorescence intensity (MFI) of each molecule, and data were presented graphically for myeloid (SSC^high^) and lymphoid (SSC^low^) cells of cranial and caudal lung tissue. * indicates a significant difference between the two groups (*p* < 0.05).

The comparison of mean fluorescence intensity (MFI) of CD18 on lung myeloid and lymphoid cells revealed a significantly (*p* < 0.05) higher abundance (4.5 times) of CD18 on myeloid cells (45,033 ± 6499 MFI) compared to lymphoid cells (9641 ± 750 MFI) (Figure 5A). Similarly, the cell adhesion molecule CD11a was significantly (*p* < 0.05) higher (2.7 times) expressed on myeloid cells (6855 ± 1019 MFI) compared to lymphoid cells (2451 ± 526 MFI) (Figure 5B). For both CD18 and CD11a, the expression level on myeloid cells and lymphoid cells was comparable (*p* > 0.05) between the cranial and caudal lung tissue (Figure 5A,B).

### 3.5. The Antibacterial Activity of Lung Phagocytes

**Figure 6:**
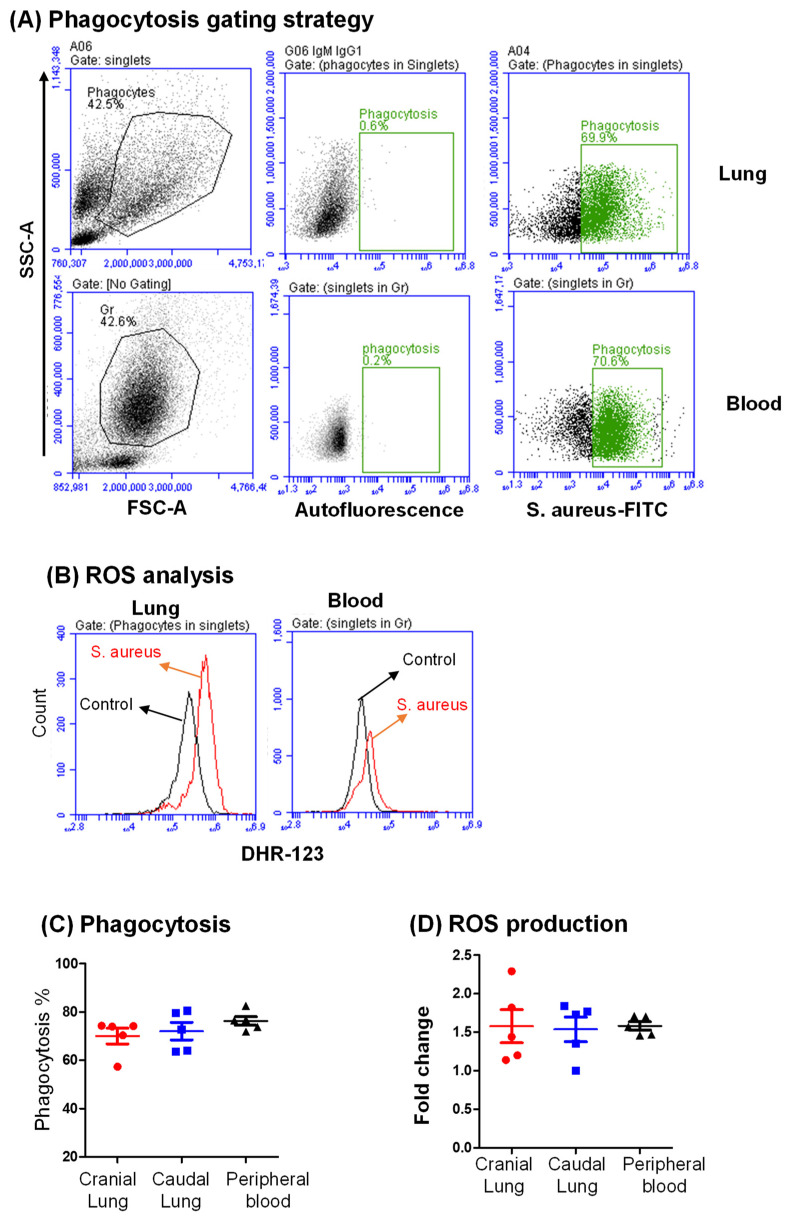
Analysis of the percentage of phagocytic cells and ROS generation by camel lung and blood phagocytes. (**A**) Analysis of phagocytosis by flow cytometry: camel lung cells or blood leukocytes were incubated with FITC-labeled *S. aureus* and analyzed by flow cytometry. After setting a gate on phagocytes (SSC^high^FSC^high^ cells) and blood granulocytes (SSC^high^FSC^low^ cells), the percentage of phagocytic cells was identified based on the elevated fluorescence in the FITC channel. (**B**) Analysis of ROS production by flow cytometry: Camel lung or blood leukocytes were stimulated with *S. aureus* bacteria or left without stimulation. ROS production was detected after the addition of dehydrorohdamin-123 (DHR-123) by flow cytometry. The amount of ROS production was evaluated based on the increased fluorescence in the FL1 channel (DHR-123 MFI) (representative overlapping histograms from one animal). (**C**) The percentage of phagocytic cells within cranial and caudal lung phagocytes or blood granulocytes was presented graphically. (**D**) The amount of ROS production was calculated as the fold increase in the DHR-123 MFI level in stimulated cells compared to basal ROS level in unstimulated cells.

The bacterial activity of lung phagocytes was evaluated by the in vitro analysis of phagocytosis (Figure 6A) and reactive oxygen species production (Figure 6B). Lung phagocytes (SSC^high^ cells) were compared with blood granulocytes. Cell incubation with FITC-labeled *S. aureus* resulted in 71.1 ± 3.2% phagocytic cells within lung phagocytes, which was slightly (*p* > 0.05) lower than the percentage of phagocytic cells within blood granulocytes (78.3 ± 2.2% of blood granulocytes) (Figure 6C). In comparison to the basal ROS level in the unstimulated cells, cell stimulation with *S. aureus* induced a comparable (*p* > 0.05) increase in ROS production in lung phagocytes (1.6 ± 0.2-fold increase) and blood granulocytes (1.6 ± 0.1-fold increase). There was no significant difference in the stimulation-induced ROS production amount between cranial and caudal lung tissue (Figure 6D).

## 4. Discussion

Respiratory tract infections are among the most common infections in dromedary camels, with a high impact on animal health, production, and welfare. Understanding the immune mechanisms in the respiratory tract is essential for the development of vaccination and therapeutic strategies for respiratory diseases [37]. The identification of immune cell populations is essential for understanding cell-specific functional roles during the immune response to infection [2]. Some important characteristics of the respiratory immune system have been investigated in some recent studies. This mainly includes the expression of the polymeric immunoglobulin receptor [38] in Bactrian camel lungs. In addition, two recent works characterized the anatomical distribution of the bronchus-associated lymphoid tissue in Bactrian camel [37] and dromedary camel lungs [39]. However, a flow cytometry procedure for the analysis of leukocyte populations in camel lung tissue is currently lacking. In the present study, fluorescent antibodies to the cell marker antigens CD45, CD172a, CD14, CD163, MHCII molecules, CD18, and CD11a were used to study some immune cell compositions in the lung tissue of dromedary camels by flow cytometry. We compared the marker-specific staining with isotype controls to estimate the fraction of cells positive for each antibody. In addition, parallel staining of peripheral blood leukocytes with the same combinations of antibodies was used for confirming the cell population bound by the antibody. In general, the higher autofluorescence of cells from the lung tissue compared to blood leukocytes resulted in an overall lower staining signal than for blood leukocytes, a phenomena that was also observed for cells in the bovine [35] and equine [27] respiratory tract.

The signal regulatory protein α (SIRP α), which is also known as CD172a, is a molecule with an inhibitory function expressed on all cells of the myeloid lineage, including macrophages, neutrophils, and monocytes [40,41]. In the present study, combined staining with CD172a and the pan-leukocyte marker CD45 identified lung myeloid cells as CD45^+^CD172a^+^ and lung lymphoid cells as CD45^+^CD172a^−^. The lower frequency of cells of the myeloid lineage with more lymphoid cells in the camel lung tissue than in the camel peripheral blood seems in contrast to the bovine system, where the lung tissue mainly comprised cells of the myeloid lineage, with only a few lymphocytes when compared to blood [24].

Based on their differential expression of the LPS receptor CD14 [18], camel macrophages (CD172a^+^CD14^high^ cells) were identified as the most abundant immune cell population in the camel lung tissue, which seems in line with their frequency in bovine and equine lungs. In contrast to their dominance in camel peripheral blood [41], granulocytes (CD172a^+^CD14^low^) presented only a minor population in the lung tissue.

MHC II molecules are antigen receptors involved in the presentation of exogenous peptide antigens to the T cell receptor and the subsequent activation of antigen-specific T helper cells [20,21]. MHCII molecules, together with CD163, the receptor for the binding and uptake of hemoglobin-haptoglobin complexes [19], are important markers of macrophage polarization [42,43]. Both molecules identified a higher cell population in the camel lung tissue than in peripheral blood. The higher frequency of MHCII-positive cells compared to CD163-positive cells can be interpreted by the different spectrum of cells expressing the two molecules, with MHCII being expressed on dendritic cells and B cells [44,45,46] in addition to macrophages, while CD163 is exclusively expressed by macrophages and monocytes [47,48,49,50].

γδ T cells are tissue-resident immune cells with a higher abundance in mucosal and epithelial surfaces, including the skin, respiratory, digestive, and reproductive tracts [51,52]. They are non-conventional T cells with a role in MHC-independent recognition of antigens, therefore bridging innate and adaptive immune responses. In the human lung, the fraction of γδ T cells represents 8–20% of pulmonary lymphocytes with an essential role in the immune response to mucosal pathogens [51]. In the camel blood and lung tissue, a similar percentage of helper T cells was identified, while γδ T cells were found at a higher frequency in the lung tissue than in peripheral blood, suggesting a role for these cells in the mucosal immune system of the respiratory tract. In the bovine lung, several subpopulations of γδ T cells were identified as having a selective role in the immune response to intracellular pathogens [25]. The same study identified a selective recruitment of the IFNg-producing WC1.1-positive γδ T cell subset to the lung tissues following intranasal BCG vaccination [25]. Whether different subsets of γδ T cells exist in the lung tissue of dromedary camels is still to be determined in future studies.

The ecuritment of blood leukocytes to peripheral tissues depends on the expression of distinct adhesion molecules and chemokine receptors, which interact with the corresponding ligands expressed by endothelial cells at the inflammation site [53]. The alpha (CD11a) and beta (CD18) chains of the integrin leukocyte-function-associated antigen-1 (LFA-1), which is expressed on several immune cell types including myeloid and lymphoid cells [54,55], were identified as the most important cell adhesion molecule involved in the recruitment of leukocyte from blood and other tissue compartments to the inflamed lungs [56,57,58]. In the present study, a higher abundance of CD11a and CD18 was found on camel lung myeloid cells compared to lymphoid cells. Further studies are required to investigate the changes in adhesion molecule expression patterns of lung immune cells during respiratory tract infection.

Pulmonary phagocytes, which mainly consist of lung macrophages and polymorphonuclear phagocytes (granulocytes) [59], are essential players in the innate immune defense of the respiratory tract [60,61]. Bacterial phagocytosis and the production of reactive oxygen species (ROS) are key effector strategies for the early clearance of pathogens by pulmonary phagocytes [62]. In the present study, flow cytometric analysis of bacterial phagocytosis and ROS production upon bacterial stimulation revealed high antimicrobial activity of camel lung phagocytes, which was comparable with the antimicrobial activity of blood granulocytes. Further studies can be planned using this methodology to investigate the antimicrobial functions of camel lung phagocytes under inflammatory conditions (infection or injury). Although dihydrorhodamine is among the most frequently used dyes for measuring ROS by flow cytometry [63,64,65], the method is limited by the reduced sensitivity to ROS and non-specific reaction of DHR with other oxidants [65]. Therefore, DHR may be replaced with other stains, such as DCFDA, in future studies.

The tissue-specific distribution of immune cells is an important factor, with an impact on the nature and outcome of the immune response to pathogens. The cranial lobe of the lung represents a primary infection site, where inhaled pathogens try to colonize and establish an infection in the lung [66]. In the present study, although the distribution of myeloid and lymphoid cells did not differ between the cranial and caudal lobes of the camel lung, the cranial lung tissue contained a higher frequency of granulocytes and a lower frequency of macrophages when compared to the caudal lung tissue. Although not proven per the functional analysis of key functions of macrophage subtypes (cytokine/chemokine production), the higher frequency of cells expressing the M2 macrophage marker CD163 in the caudal lung tissue with a slightly higher fraction of MHCII-positive cells (M1 phenotype) in the cranial lung tissue may indicate the distribution of different macrophage subtypes in the different lobes of camel lung. Such differences between lung lobes could influence the effectiveness of the immune response to infection or vaccination with respiratory pathogens. In the lung tissue of healthy human and mice, the sub-classification of macrophages into different subtypes could not be carried out based on the current macrophage classification system [67,68]. It was mainly based on their specific functions during inflammation or through driving them to a particular subtype by in vitro stimulation with recombinant cytokines [69]. Such studies are still lacking for the dromedary camel and could be a potential area of research.

Although the present study identified some subpopulations of myeloid and lymphoid cells in the camel lung, further studies are required for comprehensive immunophenotyping of the cellular pulmonary immune system in camels. Such studies require the identification of monoclonal antibodies to camel myeloid (CD11C, CD16, CD64, CD32, CD169) and lymphoid (CD3, CD8, CD2, CD25, NK cell-marker) marker antigens. In addition, the characterization of camel lung epithelial cells would allow the investigation of their cross talk with lung immune cells during the immune response to pathogens.

The results of the present study were based on the analysis of lung samples collected from five camels, which is a limitation of the current study. In addition, the tissue-specific distribution of immune cells may be affected by several physiological factors, including animal age and gender [24,70,71]. Although the selected animals were non-pregnant and non-lactating female dromedary camels, we could not exclude an impact of the reproductive cycle on the immune cell composition in the lung tissue. Therefore, further studies are required to employ the current protocol to investigate a higher number of animals from different ages, genders, and reproductive stages.

## 5. Conclusions and Perspective

The present study employed cell staining with fluorochrome-labeled antibodies and flow cytometry for the analysis of immune cell composition and function in the lung tissue of healthy dromedary camels. Some similarities and differences were identified between camels and other farm animals regarding the distribution of the main immune cell populations in their lungs. Further studies are required using more phenotypic and functional immune cell markers to investigate the changes in immune cell composition, phenotype, and function in the lung tissue of dromedary camels under inflammatory conditions (infection or injury). Dromedary camels are considered the only confirmed reservoir for the lethal Middle East Respiratory Syndrome (MERS) coronavirus and the source of zoonotic infection in humans [72,73,74,75,76,77,78,79,80]. While MERS-CoV infection in humans is associated with a severe respiratory disease with high mortality rate, MERS-CoV-infected camels show only mild and transient respiratory symptoms [81]. The protocol described in the present study may be employed to investigate the local cellular immunity in the respiratory tract of MERS-CoV-infected and recovered camels. Such studies would improve our understanding of the host-pathogen interaction mechanisms associated with the higher resistance to infection in camels, compared to humans.

## Figures and Tables

**Table 1:** List of antibodies.

Antigen	Antibody Clone	Labeling	Source	Isotype
CD14	Tuk4	PerCP	Thermofisher	Mouse IgG1
MHCII	TH81A5	-	Kingfisher	Mouse IgG2a
CD172a	DH59b		WSU	Mouse IgG1
CD163	LND68A	-	Kingfisher	Mouse IgG1
CD4	GC50A1	-	Xceltis	Mouse IgM
WC1	BAQ128A	-	Xceltis	Mouse IgG1
CD11a	HUH73A	-	WSU	Mouse IgG1
Mouse IgM	poly	APC	Thermofisher	Goat IgG
Mouse IgG1	poly	FITC	Thermofisher	Goat IgG
Mouse IgG2a	poly	PE	Thermofisher	Goat IgG

WSU: Washington State University, MHC: Major Histocompatibility Complex, WC1: workshop cluster 1, APC: Allophycocyanin, FITC: Fluorescein isothiocyanate, PE: Phycoerythrin; poly: polyclonal.

## Data Availability

The datasets used and/or analyzed during the current study are available from the corresponding author on reasonable request.
